# Internode morphometrics and allometry of Tonkin Cane *Pseudosasa amabilis*

**DOI:** 10.1002/ece3.3483

**Published:** 2017-10-16

**Authors:** Liang Cheng, Cang Hui, Gadi V. P. Reddy, Yu‐Long Ding, Pei‐Jian Shi

**Affiliations:** ^1^ Department of New Energy Science and Technology Bamboo Research Institute Nanjing Forestry University Nanjing Jiangsu China; ^2^ Centre for Invasion Biology Department of Mathematical Sciences African Institute for Mathematical Sciences Stellenbosch University Matieland South Africa; ^3^ Western Triangle Agricultural Research Centre Montana State University Conrad MT USA

**Keywords:** allometric scaling, bamboo, dimension, energy distribution, linear regression, Taylor's power law

## Abstract

*Pseudosasa amabilis* (McClure) (Poales: Gramineae) is a typical bamboo species naturally distributed in large area of south China and famous for its culm strength. Although bamboos were found to share the same development rule, the detailed internode morphology of bamboo culm was actually not fully expressed. We explored internode morphology of *P. amabilis* using 11 different physical parameters in different dimensions (1–4). As Taylor's power law (TPL) is generally applicable to describe relationship between mean and variance of population density, here we used TPL to evaluate the differences between internodes, and further, the relationship between dimension and TPL. Results showed that length (L), hollow radius (HR), hollow area (HA), hollow cylinder volume (HCV), total cylinder volume (TCV), density (De), and weight (W) all presented positive skewed distribution in varying degrees. For the basic one‐dimensional parameters, the 9th internode was the longest, the 7th the heaviest, while thickness (T) decreased with internodes. Diameter (D) decreased in general but with an inconspicuous local mode at the 5–6th internodes, potentially due to the rapid height growth. The longest (9th) internode was the “turning point” for T‐D and HR‐D relationships. Scatter plot changing trends of W to the one‐dimensional parameters after the heaviest (7th) internode were reversed, indicating a deceleration of growth speed. TPL was not holding well in one‐dimensional parameters (*R* ^2^: 0.5413–0.8125), but keep increasing as the parameter's dimension increasing (*R* ^2 ^> 0.92 for two‐dimensional, *R* ^2 ^> 0.97 for three‐dimensional, and *R* ^2 ^> 0.99 for four‐dimensional parameters.), suggesting an emergence mechanism of TPL related to both the physical dimensions of morphological measures and the allometric growth of bamboo. From the physical fundamental level, all existences are the expression of energy distribution in different dimensions, implying a more general rule that energy distribution holds better TPL in higher dimension level.

## 1 INTRODUCTION

Bamboos belong to the grass family of Gramineae, with more than 1,250 species in 75 genera, naturally distributed across the world except Europe (Scurlock, Dayton, & Hames, 2000; Jiang, 2007). Known as woody grass, nearly all bamboo species have similar chemical compositions to woody materials and thus physically much stronger than grasses (Liese, 1987; Vena, Brienzo, Garcia‐Aparicio, Goergens, & Rypstra, 2013; Liese & Kohl, 2015). Bamboos also grow the fastest among plant taxa. During the primary growth period (also known as the shooting period or height growth period), a bamboo can reach its full height (several to 30 m) within 2–4 months depending on species (Liese, 1989). Such fast growth requires the bamboo culm to possess excellent physical strength to support its upright stem structure and rapid biomass build‐up. Along the vertical bamboo culm, there are multiple hollow internodes that are separated by solid nodes, making the structure of bamboo culm a unique plant morphological trait. With the development of genomics, the growth mechanism of bamboo culms at the genetic level has started to attract attention (Wei et al., 2016). However, with mounting studies at both micro and macro levels, we are still yet to appreciate the ingenuous architecture and function of bamboo culm.

Vascular bundle is the basic unit of the culm structure. A classification system was devised in 1970s for the vascular bundle types of bamboo culms that are embedded in parenchymatous ground tissue, with four basic types identified (Grosser & Liese, 1971). The nodal anatomic structure of a bamboo in comparison with the structures of internodes was also reported, indicating that the typical vascular bundle structure disappears in the bamboo node, and there are no differences between pachymorph and leptomorph bamboo species (Ding & Liese, 1995). From the study of the Moso bamboo (*Phyllostachys edulis*), the existence of nodes was shown to have a reinforcing effect on the bending strength, longitudinal shearing strength, and compressive strength of the mechanical properties of the culm (Shao, Zhou, Liu, Wu, & Arnaud, 2010); the density of both internodes and nodes decreased from exterior to interior surface (Huang, Chang, Ansell, Chew, & Shea, 2015).

Macroscopically, the structure of a bamboo culm can be described with some basic physical parameters, such as length, diameter, and thickness. The culm structure and its variation during height growth were studied for Moso bamboos (Zhou, 1998). The thickness of bamboo culm for Moso bamboos and *Bambusa pervariabilis* is directly proportional to its diameter (Lo, Cui, & Leung, 2004). Moisture content constitutes a significant part of culm weight and declines along the culm of *Gigantochloa scortechinii* and *Bambusa vulgaris*, which can affect the dimensional stability and the strength of bamboo culm (Anokye et al., 2014). The relationship between the solid volume and green weight of the culm for *Melocanna baccifera* is linear (Singnar, Nath, & Das, 2015). Moreover, many studies have focused on the morphological characteristics of shooting and growth rhythm for specific species, such as *Bambusa wenchouensis* (Su et al., 2006), *Dendrocalamus latiflorus* (Zhou, 1999), *Bambusa distegia* (Zhang, Dong, Zhu, Zhu, & Sheng, 2011), *Dendrocalamus hamiltonii* (Li et al., 2010), and *Bashania fargesii* (Tian, 1989). However, most of these studies treated the entire culm of a bamboo as one sample, rather than a single internode. Accordingly, the discussed morphological parameters were often the total culm height and diameter at breast height (Inoue, 2013), which are meaningful at the forestry scale, but ignoring the length or diameter of each internode which contains much more detailed information for the culm morphology.

Progress in morphometrics relies on sufficient data accumulation and mathematical statistics of the individual variation for testing samples. To this end, Taylor's power law (TPL), which describes the relationship between the mean and variance of the population density (Taylor, 1961; Anderson, Gordon, Crawley, & Hassell, 1982; Shi, Sandhu, & Reddy, 2016), has been established, Variance* = a*·Mean^*b*^, where *a* and *b* are constants and *b* usually ranges from 1 to 2. TPL is a widely verified pattern in botany, ecology, entomology, and other disciplines (Taylor & Taylor, 1977; Cohen & Xu, 2015; Shi et al., 2017); however, the related TPL research on plant development and growth has attracted little attention (Picard & Favier, 2011), and the research about bamboo remains vacant. Besides, a general model for the structure and allometry of plant vascular systems was proposed based on the application of a general theory of resource distribution through hierarchical branching networks (West, Brown, & Enquist, 1999).

*Pseudosasa amabilis* McClure, commonly known as the Tokin Cane, is a native bamboo species naturally distributed in large areas of southern China, typically growing over hilly plains and hillsides along rivers (Chinese Academy of Science, 1996). *P. amabilis* has even greater mechanical strength than Moso bamboos and thus has been traditionally used for making rods and struts. In Guangdong province, an industry has been established for the utilization of *P. amabilis* culms (Yun, Tu, Ou, & Guo, 2013). The growth rhythm, basic biological characters, and biomass distribution models have already been reported (Dai, 2002; Zheng, Cao, Xiao, Chen, & Dai, 2001), whereas the changing morphology of internodes along the culm of *P*. *amabilis* is yet to be analyzed.

In this study, the internode morphology of *P. amabilis* was investigated in detail by using 11 different physical parameters in different physical dimensions: four one‐dimensional parameters representing length, three two‐dimensional parameters representing area, two three‐dimensional parameters representing volume, and two four‐dimensional parameters representing weight, respectively. Specifically, TPL was used in the statistical analysis to investigate the relationship between mean and variance of the morphological parameters of bamboo internodes, and the relationship between TPL and dimension of the selected parameters. According to this, we also tried to explore the connections between TPL, dimension, energy, and mass from the very basic point of view of physical existence.

## 2 MATERIALS AND METHODS

The bamboo species studied here is *P. amabilis*. Samples were collected and measured in a bamboo garden in Jiangdu, Jiangsu province, China (32°29′N and 119°3′E, 6 m above sea level). The climate of Jiangdu is humid subtropical, with an annual average rainfall of 978.7 mm and an annual average temperature of 14.9°C (Liu et al., 2014). The soil type is alkaline‐earth‐element‐enriched (Liao et al., 2011).

**Figure 1:**
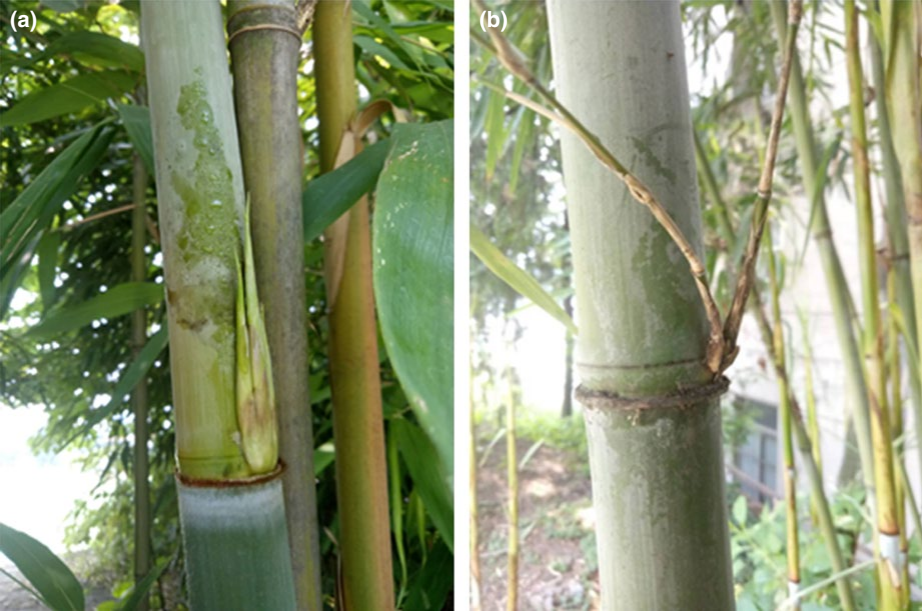
Clum and branches of *Pseudosasa amabilis*. (a) Young branch from a height growth finished new culm; (b) Adult branch from old culm

We randomly collected the culms of 30 healthy, matured (1–3 years old) *P. amabilis* individuals scattered in the 10‐acre bamboo garden area. The culms were cut from the first node position above the ground (usually also the first node without adventitious roots). The height growth period of bamboo culm is just the primary growth period and only last for a short time (30–40 days for *P. amabilis* (Dai, 2002)), and the height growth of bamboo culm is finished without branches and leaves. Meanwhile, the branches and leaves are grown from bamboo nodes (Figure 1). Thus indicating a difference of growth mechanism between culm and branches (leaves). Therefore, the branches and leaves were removed in our study about the bamboo culm and its internode. Because the internodes of *P. amabilis* at the top culm (after the 25th internode) were extremely small and often blurred together with the branches and leaves, we only counted the first to the 25th internodes in this study.

**Figure 2:**
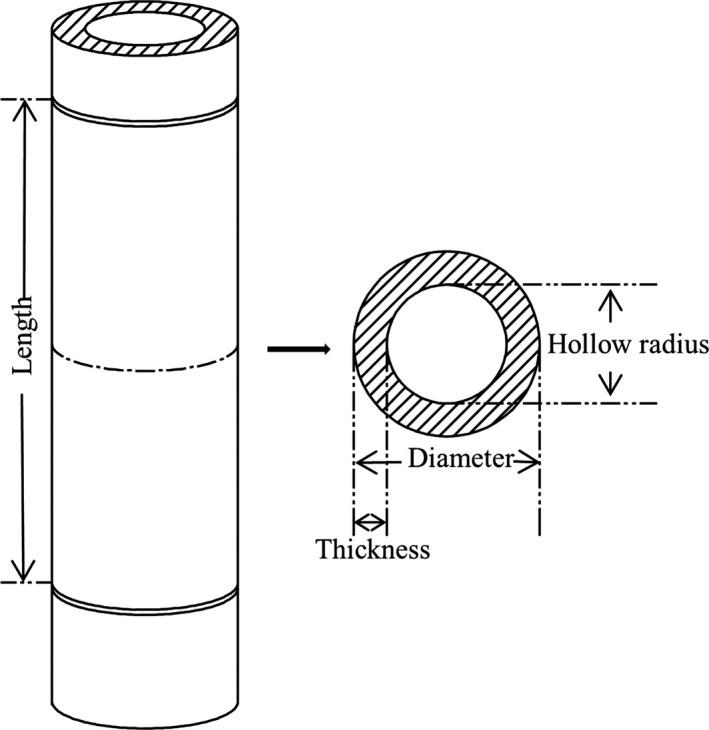
A schematic diagram of a bamboo internode

We separated each internode of a culm by using a branch scissors at each node position. From our previous investigation (Wei et al., 2016), the circle equations can obtain very approximate results for the cross‐section zone of bamboo culm. Therefore, we assumed each separated internode as a hollow cylinder (Figure 2) in this study and measured its length (L) with a tape measure, the weight (W) with a balance scale, and the diameter (D) at the middle of the internode with a vernier caliper. We then cut open the internode horizontally at the middle and measured the culm wall thickness (T) of the internode with a vernier caliper. The resolutions of the measurements were 0.1 cm for L, 0.1 g for W, 0.01 cm for D, and 0.01 cm for T, respectively.

As the impact from moisture to the morphology formation of an organism is so critical and cannot be ignored, the fresh weight is used as W in this study. In order to reduce the error caused by moisture loss, once a culm was cut down, the internodes were separated immediately and their W, L, D, and T were all measured at once.

Mathematically, D, L, and T are all basic length units in one dimension; W is considered as a four‐dimensional parameter in this study (a physical parameter in three‐dimensional space weighted by an extra dimension of material properties). The other morphological parameters of hollow radius (HR), cross‐sectional area (CSA), hollow area (HA), ring area (RA), hollow cylinder volume (HCV), total cylinder volume (TCV), and density (De) for each internode can be calculated based on the calculation formula of cylinder and these direct measures of W, L, D, and T (Table 1). The dimension of each parameter was determined based on the dimensions of L, D, T, and W, accordingly.

**Table 1:** Morphological parameters of a bamboo internode

Parameter	Abbreviation	Formulation	Dimension
Length (cm)	L	Measured	1
Diameter (cm)	D	Measured	1
Thickness (cm)	T	Measured	1
Hollow radius (cm)	HR	D/2‐T	1
Cross‐sectional area (cm^2^)	CSA	π·(D/2)^2^	2
Hollow area (cm^2^)	HA	π·(D/2‐T)^2^	2
Ring area (cm^2^)	RA	π·[(D/2)^2^‐(D/2‐T)^2^]	2
Hollow cylinder volume (cm^3^)	HCV	πL·[(D/2)^2^‐(D/2‐T)^2^]	3
Total cylinder volume (cm^3^)	TCV	πL·(D/2)^2^	3
Density (g/cm^3^)	De	W/[πL·[(D/2)^2^‐(D/2‐T)^2^]]	4
Weight (g)	W	Measured	4

We made scatter plots of: (1) morphology parameters to the internode numbers; (2) T to D and HR to D; (3) the W to the one‐dimensional morphology parameters (L, D, T, and HR). The data were logarithmically transformed, and ordinary least square was used to conduct the linear fitting using the statistic software package R (version 3.2.2; R Core Team, 2015).

We calculated the mean, standard deviation, and variance of each morphological parameter for the 1st to the 25th internode. TPL was used here to depict the relationship between the mean and variance of the morphological parameters of bamboo internodes along the axial direction of the culm. The natural logarithms of measurements were used in the linear regression: ln (Variance) = *b*·ln(Mean) + ln(*a*), with ln (Variance) treated as dependent variable and ln (Mean) independent variable, and *c* = ln (*a*).

## 3 RESULTS

### 3.1 Morphometrics of internodes

**Figure 3:**
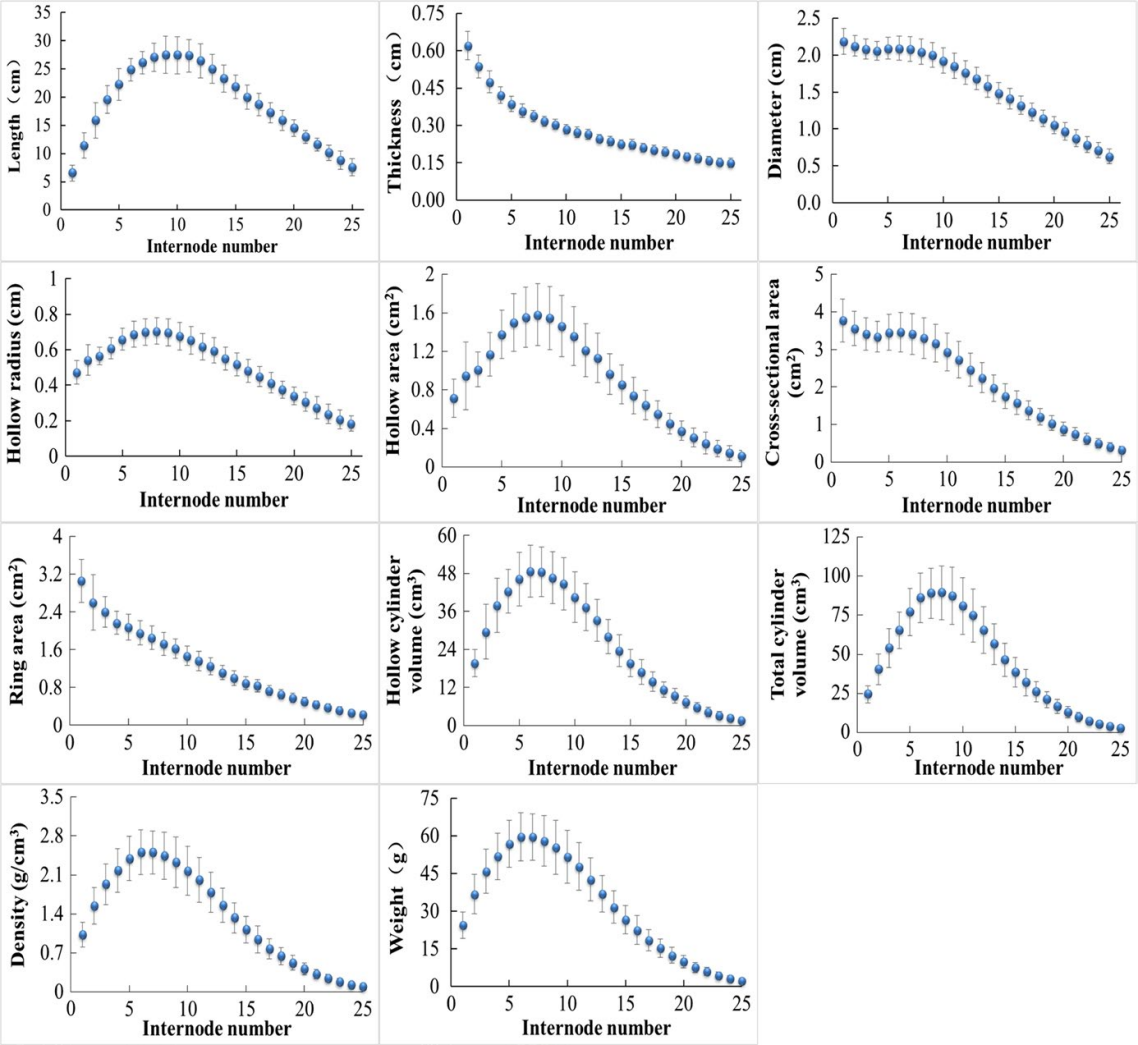
Changes in the morphology of internodes along the culm of *Pseudosasa amabilis*. Data are shown as mean ± *SD*

Generally, as internode number increasing from 1 to 25 of the bamboo culm, the morphological parameters of L, HR, HA, HCV, TCV, De, and W all presented positive skewed distribution in varying degrees; T and RA decreased continuously whereas D and CSA presented a slight increasing only for the 5th and 6th internodes because their overall changing trends were both decreasing (Figure 3).

Specifically, L increased rapidly till the 9th internode and then decreased gradually. The 9th internode of *P. amabilis* is the longest (27.52 ± 3.25 cm), the total length was 181.11 ± 14.68 cm for the 1st–9th internodes and 288.67 ± 24.44 cm for the 10th–25th internodes. The 8th internode has the biggest HR (0.71 ± 0.07 cm), HA (1.58 ± 0.32 cm^2^), and TCV (89.38 ± 17.30 cm^3^). The 6th internode has the biggest HCV (48.74 ± 8.17 cm^3^). The 7th internode has the biggest weight (59.51 ± 9.22 g) and De (2.51 ± 0.39 g/cm^3^); accordingly, the total weight of the 1st–7th internodes was 334.72 ± 53.16 g, the total weight of the 8th–25th internodes was 451.55 ± 87.09 g. From bottom to top (1st to 25th internode) of the culm, T decreased from 0.62 ± 0.06 to 0.15 ± 0.02 cm, D decreased from 2.19 ± 0.17 to 0.63 ± 0.10 cm, RA decreased from 3.06 ± 0.45 to 0.24 ± 0.05 cm^2^, and CSA decreased from 3.77 ± 0.58 to 0.32 ± 0.10 cm^2^, respectively.

**Figure 4:**
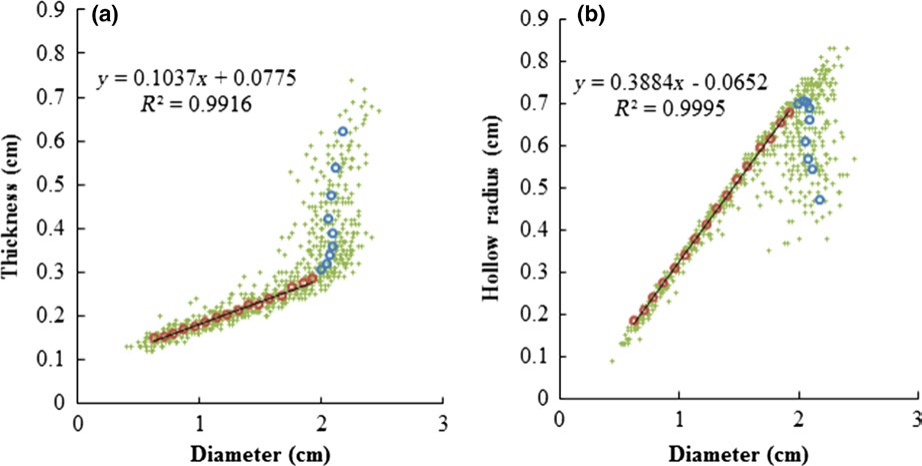
Morphometric changing of thickness, hollow radius (HR) with diameter of *Pseudosasa amabilis* culm. Green points are the raw measured data; circles are the mean, where red for 10th–25th internodes, blue for 1st–9th internodes. (a) Relationship between thickness and diameter; (b) Relationship between HR and diameter

**Figure 5:**
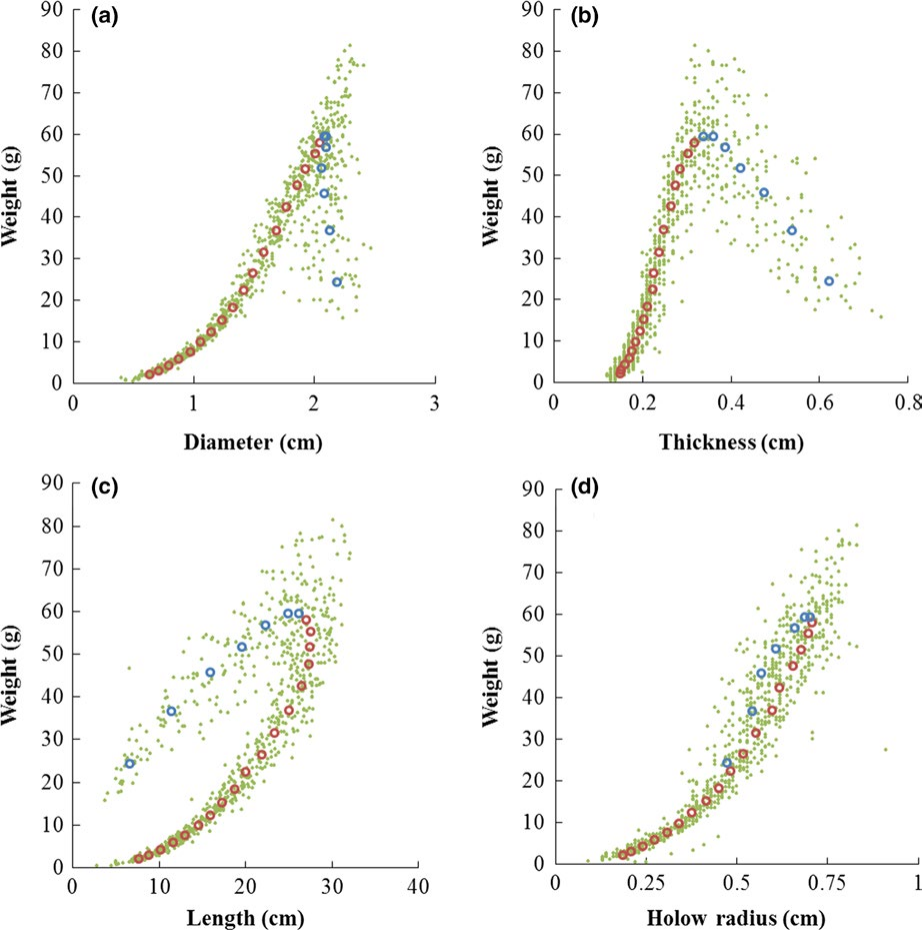
Allometric relationships between one‐dimensional parameters and weight of *Pseudosasa amabilis* internodes. Green points are the raw measured data; circles are the mean, where red for 8th–25th internodes, blue for 1st–7th internodes. (a) Relationship between weight and diameter; (b) Relationship between weight and thickness; (c) Relationship between weight and length; (d) Relationship between weight and hollow radius

D, T, and HR are the one‐dimensional parameters reflecting width. However, it seems they are somehow related to L, the changing rules of T and HR as D increasing were both divided into two different parts by the longest (the 9th) internode. When the D of an internode is less than 2 cm (>9th internode), the T–D relationship is linear (*R* ^2 ^= 0.9916); however, when D is more than 2 cm (≤9th internode), the increase of D can lead to a much fast thickening of the culm wall (Figure 4a). Compared with the T variation of 0.14 ± 0.02 cm among the 10–25th internodes, the T variation among the 1–9 internodes was much higher (0.32 ± 0.06 cm). As the HR can be calculated as HR = D/2‐T, a similar switch in the HR–T relationship can also be expected. Specifically, when D is <2 cm, the HR–D relationship is increased linearly (*R* ^2 ^= 0.9995), whereas it is going to decrease when D is more than 2 cm (Figure 4b). The HR variation among the 10–25 internodes was 0.52 ± 0.05 cm, whereas the HR variation among the 1–9 internodes was 0.23 ± 0.07 cm. With the heaviest internode (the 7th internode) as a demarcation, the W of internode at the top part followed different paths from the bottom part of the culm with the changes of one‐dimensional parameters (D, T, L, and H) (Figure 5). Specifically, W increased as D and T increased till the 7th internode, whereas W presented both increasing trends beside the 7th internode as L and HR increasing.

### 3.2 TPL analysis of the culm morphological parameters

**Figure 6:**
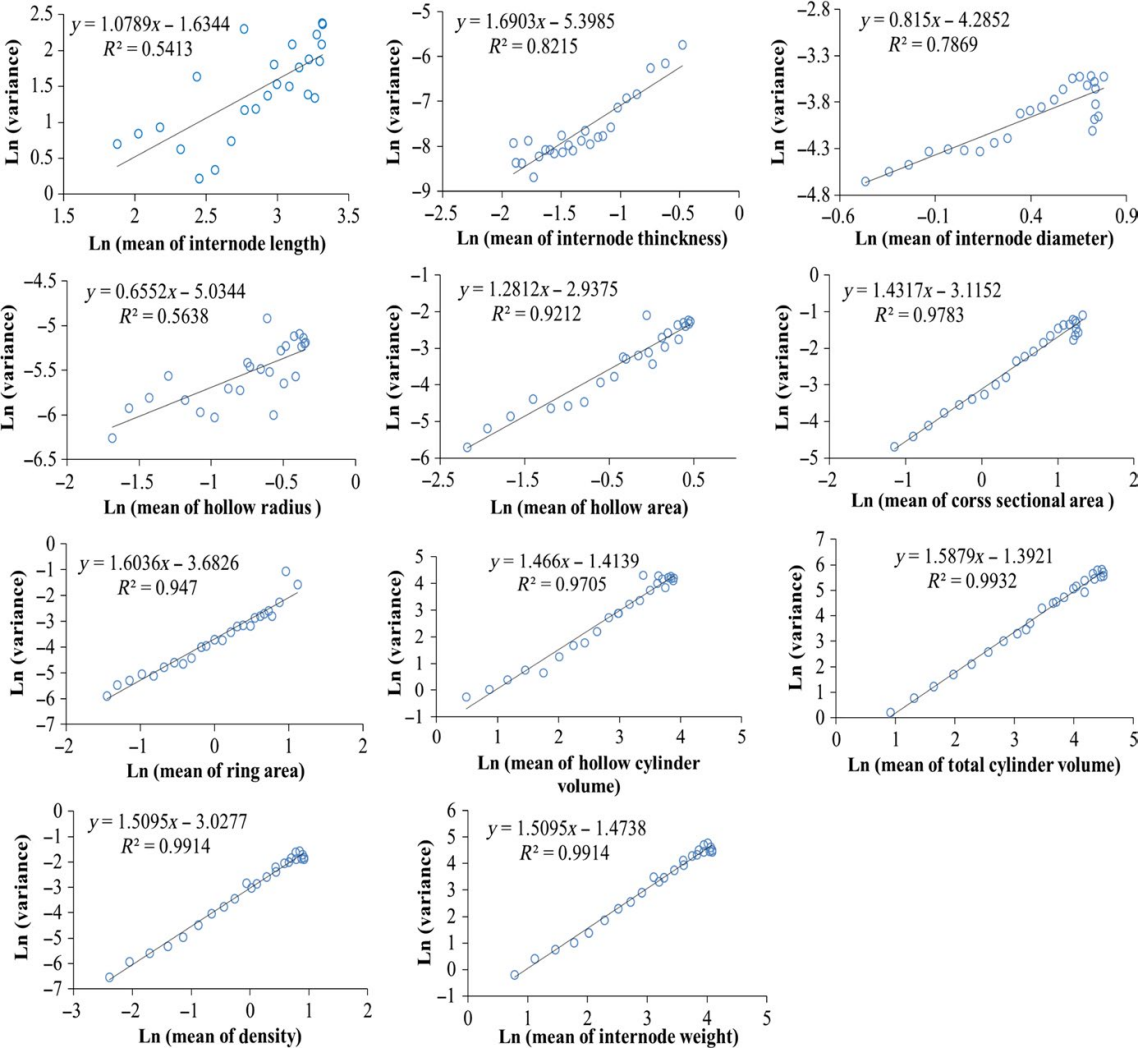
Power–law relationships between the variance and mean of morphological parameters of *Pseudosasa amabilis*

We expected the TPL for the relationship between the variance and mean of the 11 selected morphological parameters of *P. amabilis* internodes and their estimated TPL linear regression functions (Figure 6).

Evidently, among the one‐dimensional parameters, only T had a relative acceptable TPL linear regression (1 < *b *= 1.6903 < 2, *R* ^2 ^= 0.8215). Though the *R* ^2^ of D was 0.7869, its *b* was only 0.815 (<1); also the *R* ^2^ of L and HR were only 0.5413 and 0.5638, and *b* of HR was only 0.6652 (<1), therefore, this indicating that TPL was not fit for D, L, and HR.

For the two‐dimensional parameters, the *R* ^2^ improved significantly, with a corresponding *R* ^2^ order as CSA (0.9783) > RA (0.947) > HA (0.9212). For the three‐dimensional parameters, the *R* ^2^ of their TPL linear regression reached >0.97 (*R* ^2^ was 0.9705 for HCV and 0.9932 for TCV, respectively), whereas the *R* ^2^ for the four‐dimensional parameters was all above 0.99 (*R* ^2 ^= 0.9914 for both De and W). For all the two‐, three‐, and four‐dimensional parameters, their *b* was inside the range of 1–2.

## 4 DISCUSSION

For a matured bamboo culm, its primary growth has already completed; meanwhile, bamboo is monocotyledon and do not has secondary growth process (Liese, 1985). Thus, all the morphological parameters presented here of sampled bamboo culms will not change anymore. For most bamboo species, the primary growth usually takes no more than 3 months; for *P. amabilis*, it only takes 30–40 days to complete its primary height growth (Dai, 2002). This requires bamboo culm to have a corresponding enough strength to support its own body. Therefore, we found that diameter of the culm decreased from the bottom (≈2.2 cm) to the top (≈0.6 cm) internodes (Figure 3), agreed with a previous study (Liese & Kohl, 2015), but except for the 5th and 6th internodes. The diameters of the 5th and 6th internodes formed an inconspicuous local mode which might have been ignored by the previous study. From the 7th internode, the diameter declined linearly. Some bamboo species have an obvious “Z” shaped bending after the first several internodes from the bottom of the culm, such as *Phyllostachys aureosulcata* (Chinese Academy of Science, 1996). *P. aureosulcata* and *P. amabilis* are both small bamboo species, with a similar shooting period around April and May in southern China, characterized by elevated temperatures and low rainfall. Because the height growth is much faster than the lignification rate during the early period of bamboo shooting (Cheng, Adhikari, Wang, & Ding, 2015), stronger culm structures are needed to support growth and prevent bending. As *P. amabilis* is physically stronger than *P. aureosulcata*, the “Z” shaped bending in *P. aureosulcata* could have been replaced by this inconspicuous local mode in the diameter.

Compared to diameter, thickness is a one‐dimensional parameter measured about the solid part of the culm and can reflect on the strength of the bamboo culm directly. As thickness continuously decreased along the culm from bottom to the top part, this indicates that the compressive capacity of the bamboo culm increased as the node position decreased. This matches the actual situation that the bottom part is under more pressure from its own weight. Meanwhile, the natural logarithm equation was found the best form of the relationship between the culm wall thickness and the internode serial number. The corresponding ideal fitting equation is *y* = −0.126ln(*x*) + 0.5576, *R* ^2 ^= 0.9957, where *y* represents the thickness, *x* represents the internode serial number. The ratio of the total length of the 10–25th internodes to the total length of the culm is 0.614 ± 0.03, close to the Golden Ratio of 0.618 that has been widely found in natural systems (Livio, 2003). Besides this Golden Ratio of bamboo culm morphology, the only previously reported Golden Ratio of bamboo morphology is the angle between branches and culm (Wang & Zeng, 2007).

Lo et al. (2004) reported a positive linear relationship between the diameter and thickness of bamboo culms for *P. heterocycle* and *B. pervariabilis*. However, the relationship between diameter, thickness, and HR of the internodes showed a more complicated picture for *P. amabilis* (Figure 4). As diameter increased, the internode number actually decreased (Figure 3); therefore, the thickness and HR were linear increased with diameter only after the longest (9th) internode. This indicated that the 9th internode of *P. amabilis* is somehow a turning point in morphology; the upper and lower portions beside the 9th internode follow different morphological rules. Though this “turning point” at 9th internode did not appear on the relationship between thickness and HR to internode number, it clearly confirmed on the relationship between lengths to internode number (Figure 3). Physically, length, diameter, thickness, and HR are different one‐dimensional parameters; length refers to length whereas diameter, length, and HR refer to width. Therefore, as the 9th internode also the longest internode, this “turning point” at 9th internode for thickness–diameter and HR –diameter relationships should be affected by the influence from length.

The weight of each internode was the only high dimensional morphological parameter directly measured here. It is the performance of material quality (mass) under gravity. As the internode number beside the heaviest (7th) internode is not the same, the relationship between W and internode number presented a positive skewed distribution. When taking the 7th internode in the scatter plots (mean data) of weight to one‐dimensional parameters as the vertex, the scatter plot trends of weight to the one‐dimensional parameters beside the 7th internode were totally different, somehow reversed (Figure 5). Biologically, every organism has its own limit of individual size; once a correlated factor exceeds a certain range, there will be a corresponding mechanism to keep this factor within the limit. For *P. amabilis*, once the culm finished its 7th (the heaviest) internode growth during the shooting period, no matter how many internodes still remain for the following height growth, a “reverse mechanism” will be triggered for the four one‐dimensional parameters during the following growth for the deceleration of growth speed. Therefore, this ensures the culm will not be broken because the weight is beyond the limit of the culm strength due to the following growth.

Previous studies have found that many biological mechanisms hold TPL, such as animal immigration effect (Taylor & Taylor, 1977), correlation among individual reproduction (Ballantyne & Kerkhoff, 2007), and dispersal distance of plant seeds (Shi et al., 2016), etc. As a living organism, the individual sizes of bamboo culm (internode) vary in its reasonable range and share the same morphological and developmental rules (Zhou, 1998). In this study, the morphology of *P. amabilis* was expressed as 11 different parameters in different dimensions. Therefore, the goodness of fit of TPL holding for different parameters was different, as it is expressed by the *R* ^2^ of TPL linear regression directly (Figure 6). Unlike our previous expectations, TPL did not hold well for the very basic culm morphological parameters of length, HR, diameter, and thickness at first; however, this does not mean that TPL is not inappropriate for the bamboo culm morphology. As the internode parameter's dimension increased from 1 to 4, we actually can see a stepwise improving *R* ^2^ of the TPL holding clearly (Figure 6). Form the previous report (Cohen & Xu, 2015), skewness distribution can give rise to TPL, but it is not the only way TPL arises. As the parameters of length and HR can be defined as “skewness distribution,” whereas thickness, diameter, CSA, and RA cannot, the *R* ^2^ of TPL fitting of length and HR was much lower than the others; therefore, this uncertainty can be confirmed. We also found that *R* ^2^ and slope for the TPL fittings were the same for weight and density. Density can be calculated as De = W/HCV, where the dimension of W (weight) is 4, HCV is 3, respectively, and HCV can be calculated from one‐dimensional parameters (length, diameter, and thickness). Therefore, the intercept difference of the TPL fittings between weight and density was originally from length, diameter, and thickness, whereas the *R* ^2^ and slope for the TPL fittings of density follows weight, which has a higher dimension.

Some previous studies indicate that TPL might be just a general statistical pattern rather than being driven by a biological mechanism (Giometto, Formentin, Rinaldo, Cohen, & Maritan, 2015; Xiao, Locey, & White, 2015). However, from this work about bamboo culm and its internodes, a reliable correspondence between dimension(s) of the morphological parameters and the compliance degree to their TPL fittings were clearly presented. Generally, parameter with higher dimension holds better for TPL. Thus, our results suggest that the TPL is not simply a pure statistical artifact, but do related to certain physical and morphological features.

From a much broader and fundamental level of view, no matter which object is studied by using TPL, they are all physical events and must be related to mass and energy. As it is all known from Einstein's theory of Relativity, *E = mc* ^*2*^, where *E* is the equivalent energy, *m* is the mass, and *c* is the speed of light (3 × 10^8 ^m/s) (Einstein, 1905), mass actually can be considered as another expression of energy. Also, beside the known universe which has three dimensions of space and another dimension of time, the existence of extra dimensions of universe has already been proved theoretically (Li, 2005). Therefore, all the physical phenomena can be considered as different energy distribution events at different dimension level.

Realistically, energy distribution is along the entire dimensions of an event (object), this means when we working to measure an energy distribution event at a lower dimension level, the loss of information is unavoidable, and this information missing will lead to an inapplicability of TPL directly. For the bamboo internodes, weight and density mean the mass (or energy as well); all the other internode morphology parameters in lower dimensions could be considered as the measurements and expressions for energy distribution in the corresponding lower dimensions. Considering all the two‐, three‐, and four‐dimensional parameters related to thickness (thickness was included in diameter sometimes), whereas length was meaningless for the two‐dimensional measurements, this indicates a more predictive effect to weight using thickness as the only known information. Correspondingly, length was not holding TPL but thickness was close to hold TPL to some extent. For HR, it was basically not relevant to the position where weight exists, and diameter was affected by HR (D = T + HR); therefore, it is understandable that HR and diameter were not holding TPL. As another essential dimension of the universe, time is always presented as the location variations of energy (or mass) in the three‐dimensional space. Accordingly, for bamboo culm, its formation and growth actually the expressions of mass accumulation effected by time. As the TPL was clarified, population density is shown to be spatially, as well as temporally, dynamic (Taylor & Taylor, 1977); other previous studies also confirmed the space–time correlation with TPL (Lepš, 1993; Eisler, Bartos, & Kertész, 2008; Fronczak & Fronczak, 2010). This clearly explained that ideal TPL holding must be related to higher dimensions for the avoiding of information missing. From this point, it is also appropriate to say that energy distribution holds better TPL in higher dimension level.

## CONFLICT OF INTEREST

None declared.

## AUTHOR CONTRIBUTIONS

LC designed the study, carried out the experiments, and wrote the manuscript. LC and PJS analyzed the data. All the coauthors discussed and commented the manuscript.
